# Distinct cortico-striatal connections with subthalamic nucleus underlie facets of compulsivity

**DOI:** 10.1016/j.cortex.2016.12.018

**Published:** 2017-03

**Authors:** Laurel S. Morris, Kwangyeol Baek, Valerie Voon

**Affiliations:** a Department of Psychiatry, University of Cambridge, Addenbrooke's Hospital, Cambridge, United Kingdom; b Behavioural and Clinical Neuroscience Institute, University of Cambridge, Cambridge, United Kingdom; c Cambridgeshire and Peterborough NHS Foundation Trust, Cambridge, United Kingdom

**Keywords:** Compulsivity, Subthalamic nucleus, Goal-directed, Habit, OCD

## Abstract

The capacity to flexibly respond to contextual changes is crucial to adapting to a dynamic environment. Compulsivity, or behavioural inflexibility, consists of heterogeneous subtypes with overlapping yet discrete neural substrates. The subthalamic nucleus (STN) mediates the switch from automatic to controlled processing to slow, break or stop behaviour when necessary. Rodent STN lesions or inactivation are linked with perseveration or repetitive, compulsive responding. However, there are few studies examining the role of latent STN-centric neural networks and compulsive behaviour in healthy individuals. We therefore aimed to characterize the relationship between measures of compulsivity (goal-directed and habit learning, perseveration, and self-reported obsessive – compulsive symptoms) and the intrinsic resting state network of the STN. We scanned 77 healthy controls using a multi-echo resting state functional MRI sequence analyzed using independent components analysis (ME-ICA) with enhanced signal-to-noise ratio to examine small subcortical structures. Goal directed model-based behaviour was associated with higher connectivity of STN with medial orbitofrontal cortex (mOFC) and ventral striatum (VS) and more habitual model-free learning was associated with STN connectivity with hippocampus and dorsal anterior cingulate cortex (ACC). Perseveration was associated with reduced connectivity between STN and premotor cortex and finally, higher obsessive –compulsive inventory scores were associated with reduced STN connectivity with dorsolateral prefrontal cortex (PF). We highlight unique contributions of diffuse cortico-striatal functional connections with STN in dissociable measures of compulsivity. These findings are relevant to the development of potential biomarkers of treatment response in neurosurgical procedures targeting the STN for neurological and psychiatric disorders.

## 1 Introduction

The capacity to flexibly adapt to dynamic environments is a crucial component of optimal daily functioning. The development and emergence of rigid or inflexible behavioural patterns is dimensionally relevant across multiple psychiatric disorders, including addiction and obsessive-compulsive disorder. The construct compulsivity describes this tendency towards repetitive, deleterious behaviours that persist despite negative consequence (Robbins, Gillan, Smith, de Wit, & Ersche, 2012). Compulsivity can be deconstructed into several components, each detailing distinct cognitive contributions to the behavior and associated with overlapping yet distinct neural substrates.

The subthalamic nucleus (STN) is a major relay structure in the indirect pathway of the basal ganglia crucially involved in the switch between automatic and controlled processing and the balance between inhibition and executive control (Jahanshahi, 2013). The STN receives afferents from cortical regions involved in executive control (Haynes & Haber, 2013), allowing hyper-direct control of basal ganglia output based on frontal innervations. Direct cortical projections to STN, particularly from the right inferior frontal cortex (Aron, Behrens, Smith, Frank, & Poldrack, 2007) can usurp the cortico-basal ganglia loops (Balaz, Bockova, Rektorova, & Rektor, 2011) to slow, break or stop responding (Aron et al., 2007), with the STN responding to stop cues whether actions are cancelled or not (Schmidt, Leventhal, Mallet, Chen, & Berke, 2013). In rodents, STN and medial prefrontal cortex (PFC) disconnection via contralateral lesions (Chudasama, Baunez, & Robbins, 2003) and STN lesion, stimulation and inactivation (Baunez & Lardeux, 2011) enhances perseveration, a repetitive, compulsive form of responding.

Deep brain stimulation (DBS) to the STN in humans provides insight into the role of the STN in behaviour, cognition and disease states. DBS is delivered via electrodes inserted into grey or white matter and uses high frequency stimulation to modulate network activity or pathological oscillatory activity. STN DBS is effective for the symptomatic management of Parkinson's disease (PD). Impairment's in task switching in PD are improved by ventral STN DBS (but not dorsal) (Greenhouse, Gould, Houser, & Aron, 2013) implicating limbic and associative rather than motoric STN. Furthermore, STN hyperactivity in PD is associated with more habitual behaviour as measured by random number generation that requires habit suppression (Obeso et al., 2011), which is improved by STN DBS in this group (Witt et al., 2004). STN DBS targeting more limbic and associative regions has also been shown to be effective in the management of obsessive-compulsive disorder (OCD) characterized by impairments in behavioural flexibility such as enhanced habitual responding and impaired set shifting behaviours (Fineberg et al., 2015). Together these findings implicate the STN in habitual or inflexible behaviour modulation.

Recent computational models suggest parallel, interactive and dissociable systems of behavioural control: a fast, reactive and model-free system that relies on habitual learning in which previously reinforced behaviours are repeated; and a slower, deliberative model-based system for more flexible goal-directed behavior that takes into account the task-structure or internalized task model. The relative influence of each system on choice has been assessed with a two-step task, demonstrating concurrent use of both systems in healthy functioning (Daw, Gershman, Seymour, Dayan, & Dolan, 2011), and a tendency towards habitual, model-free learning in methamphetamine addiction, binge eating disorder and obsessive compulsive disorder (Voon et al., 2014). The ventral striatum (VS) has been implicated as a key node in both systems (Daw et al., 2011, Morris et al., 2015). The medial orbitofrontal cortex (mOFC) (Morris et al., 2015) and dorsolateral prefrontal cortex (dlPFC) (Smittenaar, FitzGerald, Romei, Wright, & Dolan, 2013) have been implicated in the model-based, goal-directed system. The two-step task also provides a measure of perseveration. Whereas habitual behaviours are defined as repeated choices of previously reinforced behaviours and are hence outcome sensitive, perseverative behaviors involve repetition of behaviour irrespective of the outcome. The neural correlates of perseverative behaviours are less well-understood.

Here we aimed to characterize the latent resting state network of the STN and its relationship with inter-individual variability in measures of behavioural inflexibility in healthy individuals. We hypothesize that lower goal-directed behaviours are associated with lower functional connectivity between the STN and medial OFC and dlPFC.

## 2 Materials and methods

### 2.1 Participants

Healthy volunteers were recruited from community-based advertisements in East Anglia. Psychiatric disorders were screened with the Mini International Neuropsychiatric Interview (Sheehan et al., 1998). Subjects were excluded if they had a major psychiatric disorder, substance addiction or medical illness or were on psychotropic medications. Subjects were included if they were 18 years of age or over and had no history of regular or current use of other substances.

All participants completed the National Adult Reading Test (Nelson, 1982) to assess verbal IQ. We used the self-reported Obsessive Compulsive Inventory- Revised (OCI) (Foa, Kozak, Salkovskis, Coles, & Amir, 1998) which measures subjective distress related to obsessive and compulsive thoughts and behaviours. Participants completed the behavioural measures and resting state functional MRI within the same day, with not more than 4 h of delay between. Participants provided written informed consent and were compensated for their time. The study was approved by the University of Cambridge Research Ethics Committee.

### 2.2 Tasks

#### 2.2.1 Model-free model-based task

We employed a two-step choice task (Daw et al., 2011) shown to elicit engagement of goal-directed (model-based) and habitual (model-free) learning systems, as well as perseveration (*p*). The task involved two stages. At stage 1, participants were offered a choice between two stimuli, each leading with a fixed probability to one of two states at stage 2. At stage 2, participants were offered another choice between two stimuli, each leading, with differing probabilities, to monetary reward. The probability of reward slowly shifts over the course of the task. Participants received extensive, self-paced training including practices demonstrating the concepts of stage transitions and probability, lasting 15–20 min. Choice of one stimulus at stage one led to one of two stimulus-pairs at stage two with a fixed probability (P = .70 or .30). Choice of the other stimulus led to the same stage two but with the opposite fixed probability (P = .30 or .70). Choice of a stimulus at stage two led to an independently varying probability of reward (between P = .25 to .75). Participants had 2 s to make a decision and the transition between stages was 1.5 sec. The chosen stimulus at stage one remained on the screen during stage two of that trial as a reminder. Participants completed 201 trials divided into three sessions. The outcome was an image of £1. Habit learning was modeled using a model-free reinforcement learning algorithm. However, the goal-directed learning algorithm takes into account the state transitions. A weighting factor (*w*) was calculated for each individual, capturing the relative contribution of either habitual model-free (*w* = 0) or goal-directed model-based (*w* = 1) learning. Perseveration (*p*) provides a measure of the tendency to select the same first stage choice irrespective of outcome. The task was programmed with Matlab 2011a.

### 2.3 Computational modeling

This task had three states: stage-one state A (*s*_*A*_); stage-two state B and C (*s*_*B*_ and *s*_*C*_). Each state had two actions: *a*_*A*_ and *a*_*B*_. In Model free learning was modeled using a SARSA (λ) temporal difference (TD) algorithm where each choice is based on a predicted long-run value [Q_*TD*_ (*s,a*)] for each action *a* at each stage *s*. The TD reward prediction error (δ) informs subsequent predictions. For each trial (*t*), the stage-one state s_1,*t*_ (s_A_) requires an action a_1,*t*_ choice. The stage-two state s_2,*t*_ (s_B_ or s_C_) also requires an action a_2,*t*_ choice, leading to a reward *r*_2,*t*_ (£1 or £0). After each stage *i* (1,2) of each trial *t*, a prediction error δ_*i,t*_ will occur that will update the previous states' s_*i,t*_ value Q_*TD*_ and action a_*i,t*_:

$${\text{Q}}_{\mathrm{TD}}\left(s_{i\cdot t},a_{i,t}\right)={\text{Q}}_{\mathrm{TD}}\left(s_{i\cdot t},a_{i,t}\right)+a_{i}{\text{δ}}_{\mathrm{i,t}}$$

where

$${\text{δ}}_{\mathrm{i,t}}=r_{\mathrm{i,t}}+{\text{Q}}_{\mathrm{TD}}\left(s_{i+1,t},a_{i+1,t}\right)-{\text{Q}}_{\mathrm{TD}}\left(s_{i,t}\cdot a_{i,t}\right)$$

The action value of stage-one is updated depending on the value after the stage-two state, Q_*TD*_ (*s*_2*,t*_*,a*_2,*t*_). *r*_1,*t*_ = 0 because no reward is received at this stage and r_2,*t*_ then updates the value at the second stage. The terminal value Q_*TD*_ (*s*_3*,t*_*,a*_3,*t*_) = 0. A separate parameter captures the learning rate for the update of each stage (a_1_, a_2_). The stage-one action value is updated by the stage-one prediction error and the stage-two prediction error at the end of each trial when *r*_2,*t*_ is received:

$${\text{Q}}_{\mathrm{TD}}\left(s_{1,t},a_{1,t}\right)={\text{Q}}_{\mathrm{TD}}\left(s_{1,t},a_{1,t}\right)+{\text{a}}_{1}{\text{λδ}}_{2,t}$$

This update extent is also determined by the eligibility trace parameter λ. At stage-one (*Q*_*MB*_), the model-based reinforcement learning algorithm calculated the action value per action based on the probabilities that the current action would lead to each stage two state [P(*s*_*B*_|*s*_*A*_*.a*_*A*_] = .70; [P(*s*_*B*_|*s*_*A*_*.a*_*A*_] = .30; and conversely for *s*_*C*_) and the values of those states. Therefore, for each action *a*_*j*_ (*j* = *A,B*):

$${\text{Q}}_{\mathrm{MB}}\left(s_{A},a_{j}\right)=\frac{\text{P}\left(s_{B}|s_{A},a_{j}\right){\text{maxQ}}_{\mathrm{TD}}\left(s_{B},a_{k}\right)}{k}+\frac{\text{P}\left(s_{C}|s_{A},a_{j}\right){\text{maxQ}}_{\mathrm{TD}}\left(s_{C},a_{k}\right)}{k}$$

The stage-two value is equivalent to the model-free value of the optimal action as both model-free and model-based values coincide at the end state. For each stage-one action, a net action value is calculated depending on the weighted sum of both model-free and model-based values:

$${\text{Q}}_{\mathrm{net}}\left(s_{A},a_{j}\right)=w{\text{Q}}_{\mathrm{MB}}\left(s_{A},a_{j}\right)+\left(1-w\right){\text{Q}}_{\mathrm{TD}}\left(s_{A},a_{j}\right)$$

Here, *w* is a weighting parameter and higher *w* (*w* = 1) indicates reliance on model-based learning strategies while lower *w* (*w* = 0) indicates greater reliance on model-free. At stage two, *Q*_*NET*_ = *Q*_*TD*_. For each stage, the probability of a choice is calculated using the softmax equation in *Q*_*net*_:

$$P\left(a_{i,t}=a|s_{i,t}\right)\text{α}\;\text{exp}\left({\text{β}}_{\text{i}}\left[{\text{Q}}_{\mathrm{net}}\left(s_{i,t},a\right)+p*rep\left(a\right)\right]\right)$$

where *β*_*i*_ is an index of choice reliability at each stage (β_1,_ β_2_) with higher values indicating higher reliability. *P* accounts for perseveration (P > 0) or switching (P < 0) of choices in stage one. *rep*(*a*) acts as a binary indicator such that it has a value of 1 if *a* is an action from stage one and *a* = *a*_1_,_*t−*1_, and otherwise equals 0.

### 2.4 Resting state functional MRI

We employed a novel multi-echo resting state functional magnetic resonance imaging (fMRI) acquisition and analysis with four-fold greater signal compared to noise (Kundu et al., 2012, Kundu et al., 2013), important for harnessing signal from small subcortical structures like STN. Data during rest for 10 min, with eyes open was collected with a multi-echo planar sequence using a Siemens 3T Tim Trio scanner and 32-channel head coil at the Wolfson Brain Imaging Centre, University of Cambridge (repetition time, 2.47 sec; flip angle, 78°; matrix size 64 × 64; in-plane resolution, 3.75 mm; field of view – FOV, 240 mm; 32 oblique slices, alternating slice acquisition slice thickness 3.75 mm with 10% gap; iPAT factor, 3; bandwidth = 1,698 Hz/pixel; TE = 12, 28, 44 and 60 msec). Anatomical images were also acquired with a T1-weighted magnetization prepared rapid gradient echo (MPRAGE) sequence (176 × 240 FOV; 1-mm in-plane resolution; inversion time, 1100 msec).

Functional data was denoised using multi-echo independent component analysis (ME-ICA v2.5 beta10; http://afni.nimh.nih.gov). Data were decomposed into independent components with FastICA. Blood oxygen level dependent (BOLD) percent signal change is linearly proportional to echo time (TE). Thus, independent components that strongly scaled with TE were retained as BOLD data, after assignment of high Kappa scores (Kundu et al., 2012). Components that were TE independent were measured by the pseudo-F-statistic, Rho and represent non-BOLD artefacts, which were removed by projection. This robustly denoises data for motion, physiological and scanner artefacts based on physical principles (Kundu et al., 2013). Denoised echo planar images were coregistered to their anatomical MPRAGE image and normalized to the Montreal Neurological Institute (MNI) template. For correlations with behavioural measures, but not baseline mapping, spatial smoothing was performed with a Gaussian kernel full width half maximum = 6 mm.

Functional connectivity was computed using a seed-driven approach using the CONN-fMRI Functional Connectivity toolbox (Whitfield-Gabrieli & Nieto-Castanon, 2012) for Statistical Parametric Mapping (SPM). Functional data was temporally band-pass filtered (.008 < frequency < .09 Hz). Significant principle components of white matter and cerebrospinal fluid were removed. For correlations with behavioural measures of compulsivity, STN seed-to-whole brain connectivity maps were computed and entered into second level correlation analysis controlling for age and gender. For the *w* and P scores we further controlled for the variance related to the other variable as covariates of no interest to account for multiple comparisons and highlight unique contributions of each. The STN region of interest (ROI) provided by Wake Forrest University PickAtlas (Maldjian, Laurienti, Kraft, & Burdette, 2003) was used as the STN seed. This has the same centre of mass as a previously used STN ROI based on task-based fMRI (Aron and Poldrack, 2006, Aron et al., 2007) (10, −14, −4 for right STN). Cluster extent threshold correction was used for correlations with behaviour, calculated at 15 voxels at *p* < .001 whole brain uncorrected, correcting for multiple comparisons at *p* < .05 assuming an individual-voxel Type I error of *p* = .01 (Slotnick, Moo, Segal, & Hart, 2003). Due to the possibility of mixed signals arising from adjacent structures, we also examined the adjacent substantia nigra (SN) as a seed region to ensure specificity of the current findings to STN. Thus, the same correlation for w was performed for SN-to-whole brain functional connectivity maps.

## 3 Results

### 3.1 Participant characteristics

We acquired resting state fMRI data from 77 healthy controls (46 female; age = 29.623 ± 12.168; verbal IQ = 117.133 ± 5.595; *w* = .411 ± .276; perseveration = .191 ± .173). Self reported OCI data was available for 20 of these subjects and an additional 40, totaling 60 subjects for the OCI analysis (39 female; age = 30.4 ± 12.913; verbal IQ = 115.388 ± 5.926; OCI = 10.683 ± 7.294).

### 3.2 Compulsivity measures

**Fig. 1:**
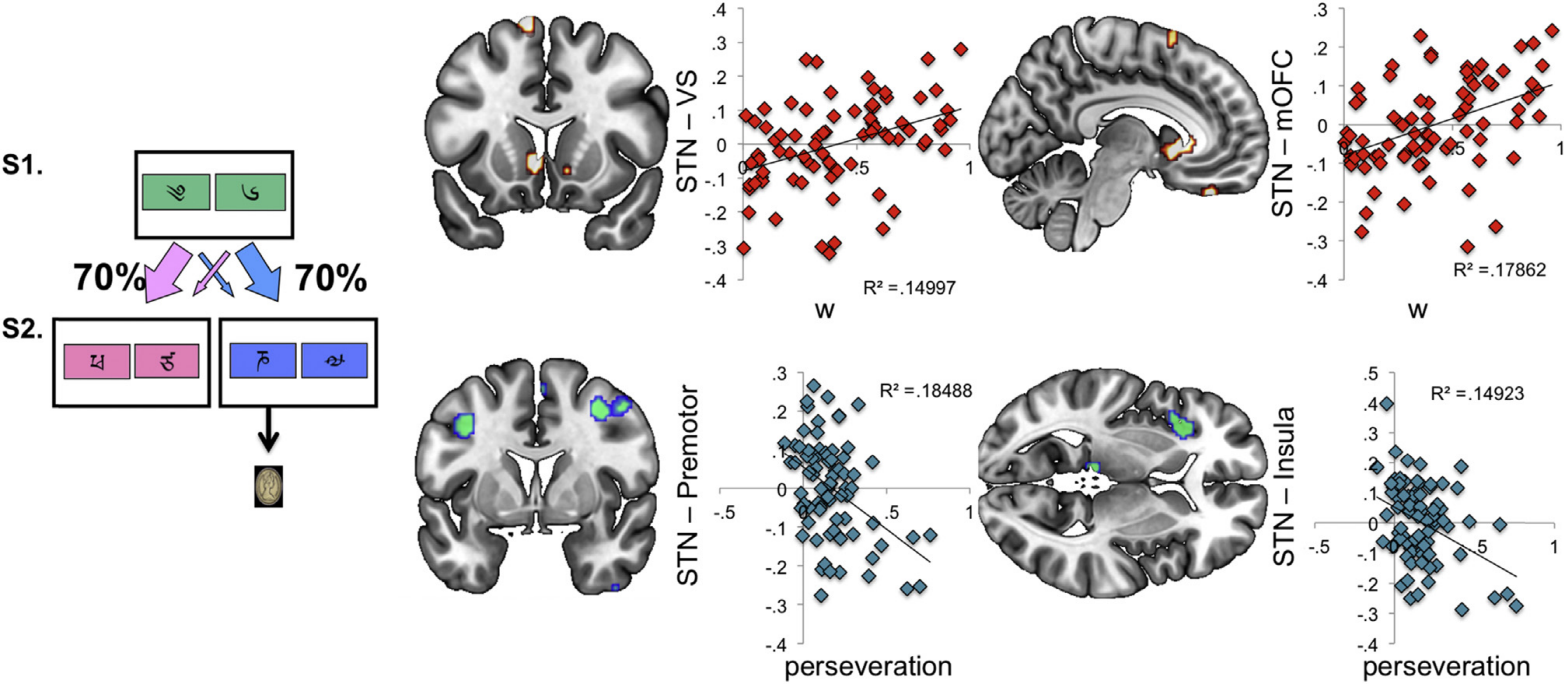
Subthalamic nucleus connectivity and model based versus model free learning. The two-step model-based model-free learning task is depicted on the left. A stimulus chosen at stage 1 (S1) led with 70/30% probability to one of two states (pink or blue in the schematic image) at stage 2 (S2). Choice of a stimulus at S2 led, with varying probability, to reward or no reward. Subthalamic nucleus (STN) connectivity with whole brain was computed and correlated with *w*, the relative contribution of model-free (*w* = 0) or model-based (*w* = 1) learning tendencies derived from the task. The *y* axis represents the functional connectivity between STN and a given region, and the *x* axis is the behavioural measure of w (top) or perseveration (bottom). STN connectivity with VS and mOFC positively correlated with w (top) and STN connectivity with premotor cortex and insula negatively correlated with perseveration (bottom). Displayed at *p* < .005 whole brain uncorrected for illustration on standard MNI template.

Table 1 demonstrates the results of the correlation between STN seed-to-whole brain functional connectivity beta maps with the measures of interest, including both positive and negative correlations. The weighting factor, w, which describes the relative contribution of either habitual (model-free, MF, *w* = 0) or goal-directed (model-based, MB, *w* = 1) learning tendencies, was positively correlated with STN connectivity with left VS and mOFC. These regions are illustrated in Fig. 1, alongside a plot of their functional connectivity with STN against w. Also, w correlated negatively with STN connectivity with left hippocampus, dorsal anterior cingulate cortex (ACC) and medial parietal cortex (statistics in Table 1).

To examine the specificity of these correlations for STN, rather than adjacent structures, we examined adjacent SN. We found no similar pattern for SN functional connectivity and its relationship with w, suggesting that the current findings for STN were not driven primarily by signals from adjacent structures (Supplementary Table 1). To further confirm this, functional connectivity for adjacent SN (with regions currently implicated for STN and w, VS, medial OFC, dorsal ACC, hippocampus) was computed and correlated with w. No significant correlations were observed between adjacent SN and regions implicated for STN, with w (see supplementary materials).

**Fig. 2:**
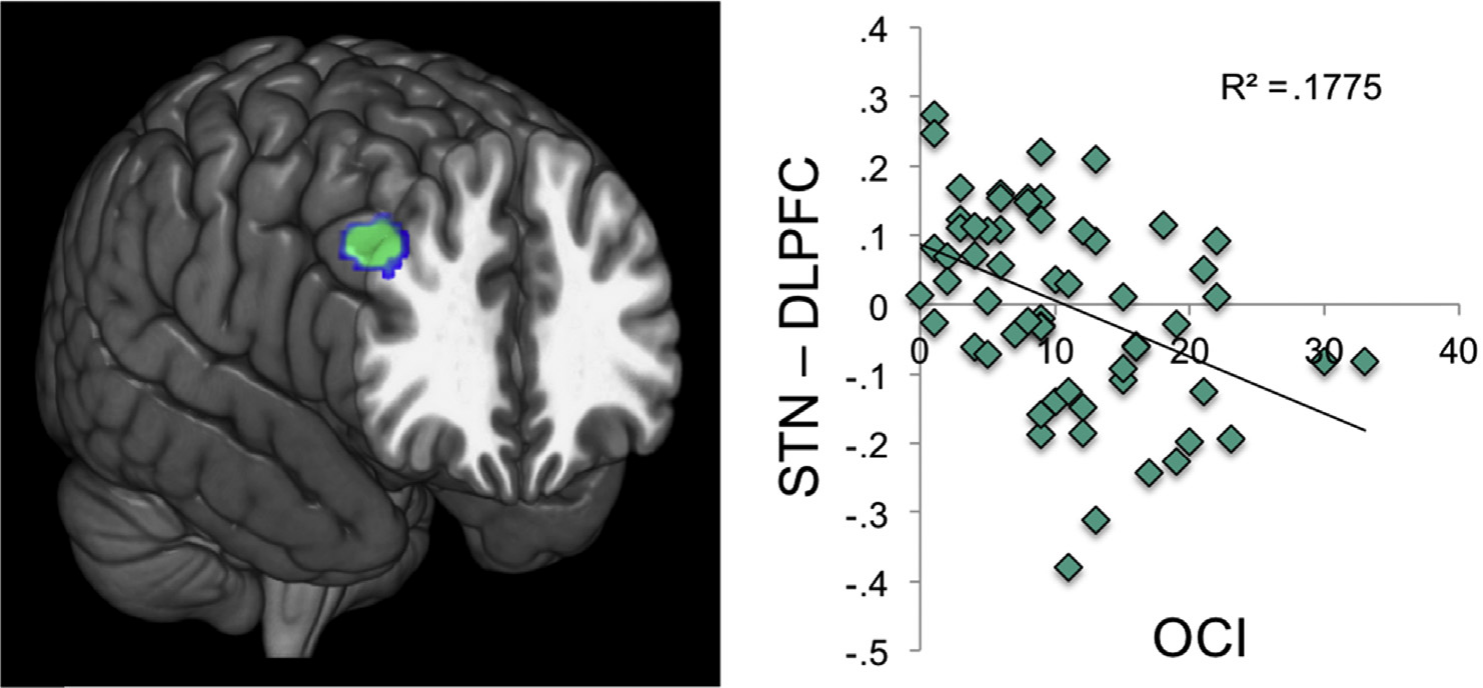
Subthalamic nucleus connectivity and compulsivity. Subthalamic nucleus (STN) connectivity with whole brain was computed and correlated with obsessive compulsive index. Abbreviation: DLPFC, dorsolateral prefrontal cortex. Displayed at *p* < .005 whole brain uncorrected for illustration on standard MNI template.

For comparison purposes we also investigated perseveration, which was associated with reduced connectivity between STN and left premotor cortex and left insula (Fig. 1). OCI score negatively correlated with connectivity between STN and cerebellum, right dlPFC and left inferior parietal cortex (Fig. 2). There were no positive correlations for OCI.

## 4 Discussion

We illustrate the relationships between intrinsic resting state functional connectivity of the STN and behavioural measures of compulsivity across a relatively large sample of healthy volunteers. Higher connectivity between STN with medial OFC and left VS was associated with more model-based goal-directed learning whereas more model-free habitual learning implicated STN connectivity with dorsal ACC and left hippocampus. Furthermore, perseveration was associated with STN with premotor and insula connectivity whereas higher self-reported obsessive compulsive scores were associated with lower connectivity between STN and right dlPFC and left inferior parietal cortex. We highlight unique neural couplings of the STN, contributing to distinct measures of compulsivity.

The relationship between model-basedness and STN connectivity with OFC and VS dovetails with several studies implicating this cortico-striatal pathway in model-based learning. Model-based behaviour has been associated with higher grey matter volume in the medial OFC (Voon et al., 2014) and the reward prediction errors used to guide both model-based and model-free behaviour are encoded by the VS (Daw et al., 2011). Furthermore, we have previously demonstrated that higher functional connectivity between medial OFC and VS is associated with greater model-based learning tendencies using the same task (Morris et al., 2015).

In contrast, greater habitual model-free learning was associated with greater connectivity of the STN with dorsal ACC and hippocampus. The neural correlates of model-freeness have been less well established. Previous studies assessing habitual behaviour in humans have implicated the putamen and premotor cortex using the ‘slips of action’ task (de Wit et al., 2012) and the supplementary motor area (SMA) using the current two-step task (Morris et al., 2015). Traditionally, there has been a dissociation between dorsal striatal habit and hippocampal declarative or cognitive memories driving behaviour (Broadbent et al., 2007, Packard et al., 1994, Wingard and Packard, 2008). However, the hippocampus has been shown to encode reward prediction (Tanaka et al., 2004), which is necessary for the reinforcement learning that drives model-free behaviour (Glascher, Daw, Dayan, & O'Doherty, 2010). The dorsal ACC receives extensive projections from dopaminergic midbrain projections and is also implicated in reward prediction and prediction error for guiding reinforcement driven behaviour (Holroyd and Yeung, 2012, Kennerley et al., 2006). Links between the STN and dorsal ACC have been exemplified by studies in PD patients, which show that STN DBS reduces cerebral blood flow in the dorsal ACC (Ballanger et al., 2009, Thobois et al., 2007). STN DBS affects habitual behaviour, as measured by the generation of a sequence of random numbers (requiring habit suppression), although DBS has been shown to both improve (Witt et al., 2004) and impair (Thobois et al., 2007) performance on this task. STN DBS has also been shown to consistently hasten responding in the context of conflict or competing responses related to mesial prefrontal theta activity (Cavanagh et al., 2011). In the context of habit learning, conflict resolution may be relevant in resolving choices that involve switching between strategies. Thus, the STN may mediate the shift between automatic habit learning from enhanced reliance on previously encoded reward prediction mediated via dorsal ACC and hippocampal structures to controlled goal-directed learning via the representation of goals in the medial OFC to flexibly guide responding.

Both w and perseveration capture similar repeated choices but are dissociated as a function of relevance of previously learned outcomes. We implicate a relationship between perseveration and STN connectivity with premotor cortex, a region responsible for action ownership and recognition (Ehrsson et al., 2004, Rizzolatti et al., 1996). Changes in perseveration for reward (Albuquerque et al., 2014, Herzog et al., 2009, Houeto et al., 2002) are observed following STN DBS in PD. Thus, whereas habit learning implicates regions involved in the encoding of reward prediction, perseveration implicates motor preparatory regions. Finally, higher obsessive-compulsive inventory scores were associated with weaker connectivity between STN and a fronto-parietal executive network including dorsolateral PFC, a network crucial for cognitive and attentional flexibility and shifting and implicated in OCD (Fineberg et al., 2015).

We chose to examine resting state neural properties rather than task-based for several reasons. Firstly, understanding the resting and latent neural network provides insight into the default or intrinsic function of the network as a whole-without perturbation by cognition, which may differ on an interindividual basis. As such, two levels of interindividual variability are possible: variability within the intrinsic network itself; and variability in the way in which that network is recruited during task. This distinction certainly requires further exploration and delineation. However, understanding the baseline characteristics of neural networks is key, before any network recruitment by task demand. Furthermore, resting state fMRI data is quicker and easier to collect compared to task fMRI- features that are crucial in clinical settings. As the current study is of relevance to clinicians interested in STN DBS, we use a tool that is accessible to clinical work. This technique can therefore be expanded to other areas of clinical interest, for example for pre-surgical mapping studies based on behavioural or cognitive faculties of particular importance. While we employ a technique that improves signal compared to noise for examining small structures, there are certainly still limitations for the use of 3T fMRI for examining such small regions, where the signal can be mixed or contaminated by adjacent structures. We aimed to combat this by illustrating that the observed findings were not produced primarily from the adjacent SN.

Together the findings highlight unique contributions of diffuse cortico-striatal functional connections with STN to dissociable measures of compulsivity. These observations are particularly relevant to the impact of STN DBS on behavioural inflexibility in neurological and psychiatric disorders and may potentially act as biomarkers of treatment response.

## Figures and Tables

**Table 1:** Statistics of subthalamic nucleus connectivity and compulsivity.

	Cluster	Z	x	y	z
**w positive**					
Bilateral ventral Striatum	65	4.35	13	24	−4
	89	4.26	−6	14	−2
Left medial OFC	29	4.22	−6	38	−30
Right temporal	29	3.76	64	−30	−23
	19	3.56	−66	−32	17
**w negative**					
Dorsal ACC	31	4.87	8	28	19
Left hippocampus	38	4.44	−31	−20	−18
Posterior Cingulate	26	4.12	−13	−23	33
Medial Parietal	34	3.92	−10	−65	49
		3.4	−8	−74	49
	21	3.66	1	−74	56
Cerebellum	19	3.63	−41	−58	−53
Midbrain	17	3.58	3	−25	−18
		3.32	8	−20	−23
**OCI positive**					
Nil					
**OCI negative**					
Cerebellum	47	3.88	−48	−65	−51
		3.78	−48	−48	−49
	32	3.8	−6	−27	−58
Left Inferior Parietal	51	3.86	−45	−46	40
Right Dorsolateral PFC	21	3.35	50	33	35
**Perseveration positive**					
Left Cerebellum	17	3.58	−8	−48	−9
**Perseveration negative**					
Left Occipital	24	4.58	−27	−76	12
Left Premotor Cortex	23	4.52	−38	3	45
Left Insula	20	4.06	−20	19	35

Statistics for the bilateral subthalamic nucleus (STN) seed-to-whole brain connectivity positive and negative correlations with measures of compulsivity. Cluster extent threshold correction of 15 voxels at *p* < .001 whole brain uncorrected was used. Abbreviations: Z, Z score; xyz, peak voxel coordinates; w, weighting of model based (*w* = 1) and model free (*w* = 0) learning; OCI, obsessive compulsive index; OFC, orbitofrontal cortex; ACC, anterior cingulate cortex; PFC, prefrontal cortex; IFC, inferior frontal cortex.
