# Supplementary material for: Distinct cortico-striatal connections with subthalamic nucleus underlie facets of compulsivity
Source: Cortex. 2017 Mar;88:143–50. doi: 10.1016/j.cortex.2016.12.018 (PMC5333782; doi:10.1016/j.cortex.2016.12.018)
Supplement: Supplementary file 1 [file mmc1.docx]

Supplementary Materials

Supplementary Results

To examine the specificity of the subthalamic nucleus (STN) findings to STN rather than adjacent structures, we examined the substantia nigra (SN). There were no overlapping regions whose connectivity with SN was correlated with w (Supplementary Table 1). To examine whether adjacent SN contributed to the current findings for STN, we computed SN connectivity with the regions and peak coordinates implicated in the main study (ventral striatum (xyz=13,24,-4), medial orbitofrontal cortex (OFC, xyz=-6,38,-30), dorsal anterior cingulate cortex (ACC, xyz=8,28,19), hippocampus (xyz=-31,-20,-18). These connectivity measures were correlated with w and no significant correlations were observed (SN and ventral striatum functional connectivity correlated with w, R=-0.018, p=0.877; SN and medial OFC, R=-0.009, p=0.937; SN and dorsal ACC, R=-0.201, p=0.078; SN and hippocampus, R=-0.134, p=0.242).

Supplementary Table 1

|  | Cluster | Z | x | y | z |
| --- | --- | --- | --- | --- | --- |
| *positive* |  |  |  |  |  |
| Occipital | 26 | 4.39 | 29 | -60 | 0 |
| Cerebellum | 30 | 4.28 | 17 | -14 | -42 |
| Inferior Temporal | 18 | 4.06 | 36 | -46 | -2 |
| Inferior Temporal | 16 | 3.83 | 64 | -34 | -25 |
| *negative* |  |  |  |  |  |
| nil |  |  |  |  |  |

Supplementary Table 1. Statistics of substantia nigra connectivity and positive and negative correlations with w. Statistics for the bilateral substantia nigra seed-to-whole brain connectivity positive and negative correlations with w (weighting of model based (w=1) and model free (w=0) learning). Cluster extent threshold correction of 15 voxels at p<0.001 whole brain uncorrected was used. Abbreviations: Z, Z score; xyz, peak voxel coordinates.
